# Trends in burden of falls among children aged 0–14 years in China from 1990–2021 and prediction to 2030

**DOI:** 10.3389/fpubh.2025.1697972

**Published:** 2025-12-19

**Authors:** Huali Xiong, Daiqiang Liu, Xiaoqin Yuan, Yue Yang

**Affiliations:** 1 Center for Disease Control and Prevention of Rongchang District, Chongqing, China; 2 The People's Hospital of Rongchang District, Chongqing, China; 3 Chongqing No. 48 Middle School, Chongqing, China; 4 Xueyuan Road Primary School, Rongchang District, Chongqing, China

**Keywords:** falls, burden, children, trend, joinpoint regression model, prediction

## Abstract

**Background:**

Although falls are the major cause of non-fatal injuries and preventable deaths among Chinese children, comprehensive assessments of the temporal trends in the burden of falls among children aged 0–14 years in China remain scarce. To address this gap, we quantified national and temporal trends in burden of falls among children aged 0–14 years from 1990 to 2021 and projected future trends through 2030.

**Methods:**

The current study used data from the Global Burden of Disease Study 2021 (GBD2021), we extracted crude incidence, mortality, years of life lost (YLLs), years lived with disability (YLDs), and disability-adjusted life years (DALYs) attributable to falls along with their corresponding absolute counts for Chinese children aged 0–14 years during 1990–2021. All metrics were stratified by sex and 5-year age group. Age-standardized rates (ASRs), including age-standardized incidence rate (ASIR), age-standardized mortality rate (ASMR), age-standardized YLLs rate, age-standardized YLDs rate, and age-standardized DALY rate (ASDR) for children aged 0–14 years, were recalculated using the World Health Organization’s standard population. Temporal trends were assessed with Joinpoint regression model to compute average annual percentage changes (AAPCs). Finally, autoregressive integrated moving average (ARIMA) models were developed to project ASRs for children aged 0–14 years through 2030.

**Results:**

Between 1990 and 2021, children aged 0–14 years experienced 163,769,426 incident cases and 255,840 deaths, resulting in a total of 25,085,796 DALYs. The ASIR, ASMR, age-standardized YLLs rate, age-standardized YLDs rate, and ASDR all demonstrated a downward trends with AAPCs of −0.34% (95%*CI*: −0.39% to −0.28%, *P*<0.001), −4.18% (95%*CI*: −4.37% to −4.06%, *p* < 0.001), −4.24% (95%*CI*: −4.43% to −4.11%, *p* < 0.001), −1.16% (95%*CI*: −1.22% to −1.11%, *p* < 0.001), −3.68% (95%*CI*: −3.80% to −3.57%, *p* < 0.001), respectively. In stratified analysis, similar downward trends were observed aross both sexes and age group of 0–4 years, 5–9 years and 10–14 years. Notably, upward trends were observed in the ASIR from 2010 to 2021 and the age-standardized YLDs rate from 2010 to 2021. Children aged 10–14 years exhibited an upward trend in incidence rate 1990 to 2021. Predictions shows the incidence among children aged 0–4 years is projected to rise, and an increase in YLDs is anticipated among children in both the 0–4 years and 5–9 years age groups.

**Conclusion:**

The burden of falls remains a major public challenge among children aged 0–14 years, although its burden at the national level showed a downward trend from 1990 to 2021. The age-standardized YLDs rate among males, the incidence among children aged 0–4 years and the YLDs among children aged 0–9 years are projected to increase from 2022 to 2030. These findings suggest that mandatory implementation of age-specific fall-prevention protocols in kindergartens and primary schools nationwide and expand the coverage of injury surveillance and implement it nationwide.

## Introduction

Falls are defined as sudden, involuntary, and unintentional changes in body position that result in contact with the ground or a lower surface. According to the World Health Organization classification, falls are divided into two main types: ① Falls from one level to another and ② Falls on the same level (1). In 2021, falls caused 802,803 deaths worldwide, corresponding to a crude mortality rate of 10.71/100,000 (2). This represents a substantial increase of 33.07% compared with the 1990 rate of 7.65/100,000. Among all age groups, motor vehicle collisions, falls, and interpersonal violence now constitute the top three external causes of death and disability globally (3). Yet when the lens is narrowed to children (3). Falls are prevalent in childhood and can be regarded as a natural component of growth and development (4). Nevertheless, their impact on children’s health should not be overlooked (3). Falls may also cause critical conditions such as complex fractures and damage to abdominal organs (5). Additionally, falls are the leading cause of injury-related hospitalization among children under 5 years (6). They also represent the most common cause of non-fatal injuries and disabilities in this population, highlighting the growing public health concern associated with childhood falls.

China offers a telling illustration of this data gap (3). Falls are the predominant cause of injury among children aged 0–14 years in China. They also contribute substantially to disability-adjusted life years (DALYs) lost in this population (7). The Chinese Cause of Death Surveillance Dataset (2018 edition) revealed that approximately 42,000 cases of falls among children aged 0–5 years were documented, representing 56.76% of all injury causes (8). Between 2010 and 2019, the mortality rate from falls among children and adolescents aged 10 to 19 years showed an increasing trend, with an average annual percentage change (AAPC) of 2.90% (9). The Review Report on the Prevalence of Injuries Among Children in China (2016–2018) indicated that falls emerged as the primary cause of injury among children from 2016 to 2018. Additionally, falls were the principal cause of emergency department visits and outpatient care for children and adolescents, accounting for 49.66% of all injury-related visits (10). In addition to causing child deaths, falls more frequently lead to non-fatal outcomes, including disability, functional limitations, and restricted mobility. These outcomes impose substantial physical and psychological burdens on children and have a profound impact on their families (11, 12). Therefore, both the loss of young lives from falls and the lifelong consequences of disability underscored the urgent need for falls prevention among children and adolescents.

While studies on fall-related burden in the general population and older adults had been gaining momentum (1, 13, 14). However, detailed analyses of the disease burden caused by falls among children aged 0–14 years in China remain conspicuously scarce. Most existing studies in the Chinese context are either limited in temporal scope, lack age- and sex-stratified analyses, or omission of forward-looking projections. This study aims to fill these critical gaps by providing a comprehensive, long-term assessment from 1990 to 2021. This study offers three principal advances. First, we employ Joinpoint regression to quantitatively delineate distinct temporal phases and turning points in the burden of childhood falls, offering a more nuanced understanding of trends than simple linear models. Second, our analysis is stratified by sex and detailed age groups (e.g., 0–4, 5–9, 10–14 years), which is crucial for identifying high-risk subgroups and informing targeted interventions. Finally, and most distinctively, we project the future burden of childhood falls up to 2030 based on the identified trends. These projections provide a forward-looking perspective that can guide the formulation of proactive, evidence-based public health policies. By achieving these objectives, this study will yield critical, previously unavailable insights into the evolving epidemiology of falls among children aged 0 to 14 years in China, thereby directly informing the development of timely and targeted prevention strategies to mitigate this significant public health concern.

## Methods

### Data source

The data utilized in the present study were derived from the GBD 2021 database. The GBD2021 systematically assessed the incidence, prevalence, mortality, and disability-adjusted life years rates for 369 diseases and injuries and 87 risk factors across 204 countries and territories from 1980 to 2021, by integrating literature resources, surveillance data, survey information, medical records, and health insurance data (15). In China, data were primarily drawn from a combination of national censuses, population-based surveys, the Disease Surveillance Points system, and the Cause of Death Reporting System managed by the Chinese CDC, and systematic reviews that evaluated disease incidence and prevalence rates (1). Specifically, we retrieved information on incident cases, deaths, years of life lost (YLLs), years lived with disability (YLDs) and disability-adjusted life years (DALYs) related to falls among children aged 0–14 years in China, along with the corresponding crude rates (per 100,000 population) and their 95% uncertainty intervals (UIs), covering the period from 1990 to 2021. The data were stratified by sex (both, males and females), age group (0–14 years, 0–4 years, 5–9 years, 10–14 years), and year using the GBD Results tool (available at https://vizhub.healthdata.org/gbd-results/).

### Statistical analysis

The age-standardized incidence rate (ASIR), age-standardized mortality rate (ASMR), age-standardized YLLs rate, age-standardized YLDs rate, and age-standardized DALY rate (ASDR) for children aged 0–14 years were recalculated using the World Health Organization 2000–2025 world standard population as the reference. The calculation process involved the following steps:

1. Determine the Proportion of age group (0–4 years, 5–9 years and 10–14 years) in the WHO Standard Population: Calculate the overall percentage of the 0–14 age group within the WHO world standard population.
2. Calculate Weighted Proportions for Each Age Subgroup: Compute the weighted proportion of each age subgroup (0–4 years, 5–9 years, and 10–14 years) within the 0–14 age range.
3. Compute Weighted Rates: Multiply the age-specific crude rates (incidence, mortality, YLLs, YLDs, and DALYs) by their corresponding weighted population proportions to derive the weighted rates for each age subgroup.
4. Sum Weighted Rates Across Age Subgroups: Aggregate the weighted incidence, mortality, YLL, YLD, and DALY rates across all age subgroups to obtain the age-standardized rates for the entire 0–14 age range. This procedure was applied to the age-specific crude incidence, mortality, YLLs, YLDs, and DALYs rates for each year and age subgroup, thereby generating the age-standardized rates for children aged 0–14 years.

The temporal trends in age-standardized rates (ASR) of falls among children aged 0–14 years from 1990 to 2021, stratified by sex and age group, were assessed with Joinpoint regression model. Annual percentage change (APC) and average annual percentage change (AAPC), as well as their respective 95% confidence intervals (CI), were computed using the Joinpoint Regression Program developed by the National Cancer Institute (available at https://surveillance.cancer.gov/joinpoint/). An upward trend was identified when both the AAPC and the lower bound of the 95%*CI* were positive values (*p* < 0.05) (16), conversely, a downward trend was determined if both the AAPC and the upper bound of the 95%*CI* were negative values (*p* < 0.05) (15). If neither of these conditions was met, the ASRs were considered to be stable.

To forecast short-term future trends in the ASRs of falls among children aged 0–14 years, the autoregressive integrated moving average (ARIMA) model was employed. This model was selected for its simplicity and well-documented effectiveness in time-series forecasting within public health research (17–19). The performance of the ARIMA model was rigorously evaluated using several statistical metrics, including root mean square error (RMSE), mean square error (MSE), mean absolute error (MAE), and mean absolute percentage error (MAPE). Lower values for these metrics are indicative of higher forecasting accuracy. Specifically, only models achieving a MAPE of less than 5% were considered to meet the acceptable standards for predictive accuracy. To further assess the adequacy of the selected model, the Ljung-Box Q-test was performed. The results indicated that the residuals approximated white noise, with a *p*-value greater than 0.05. This finding confirmed the absence of autocorrelation among the residuals, thereby supporting the model’s reliability and predictive validity for forecasting the ASRs of falls in the specified pediatric population.

Crude incidence, mortality, and YLLs, YLDs, DALYs and ASRs were computed in Excel 2013. Temporal trends of ASRs among children aged 0–14 years were assessed with Joinpoint regression model (version4.6.0.0, Applications Branch, National Cancer Institute, Bethesda, USA). Forecasting study was conducted by the auto.arima() function from the forecast package in R software (version 4.0.3). Trend changes in the proportion of YLDs relative to DALYs were analyzed by *χ*^2^ test. Figures were produced in GraphPad Prism 8.0. All tests were two-sided, with α = 0.05.

## Results

### Changing in disease burden of falls among children aged 0–14 years in China, 1990–2021

**Figure 1:**
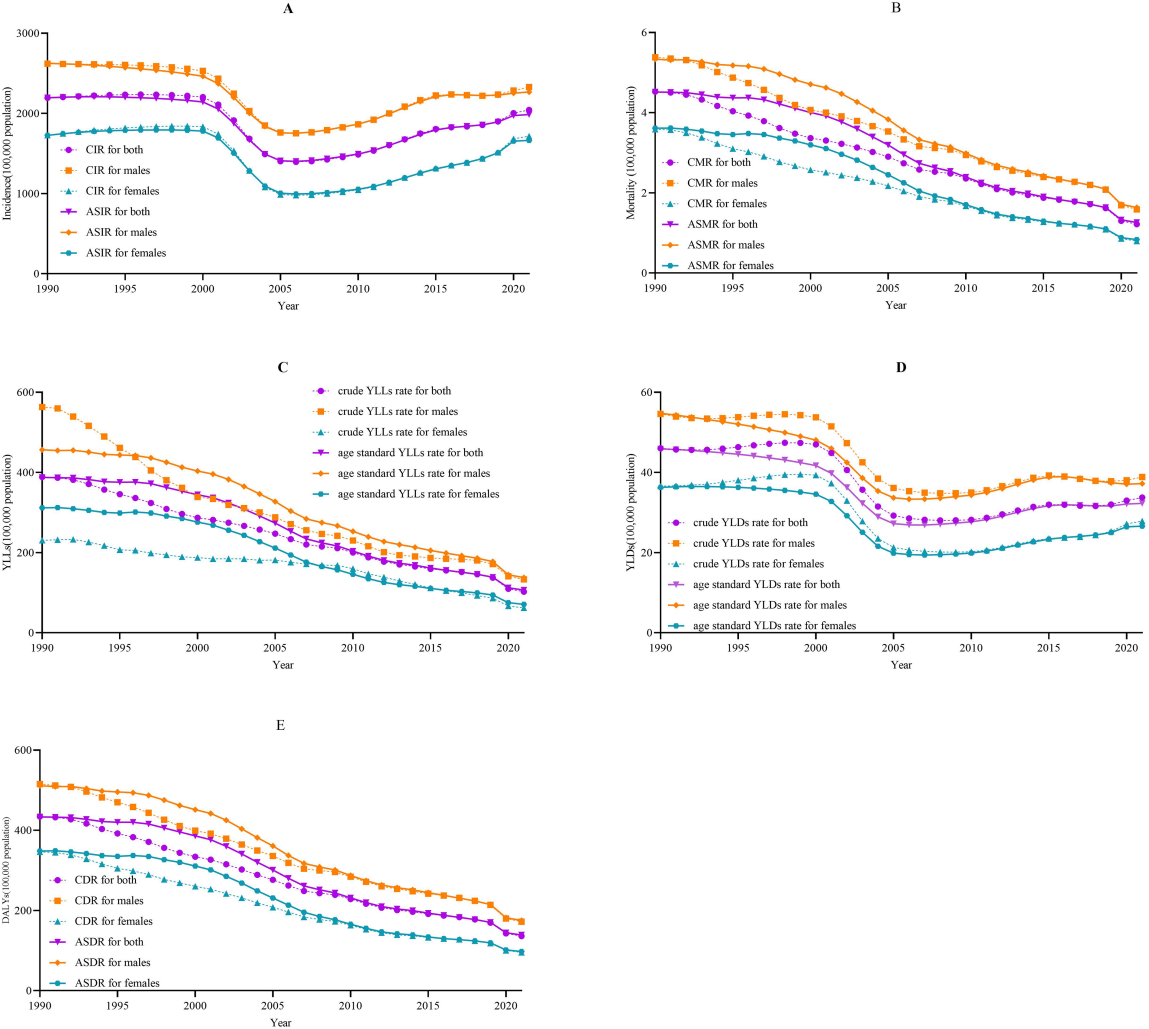
The disease burden of falls among children aged 0–14 years in China, 1990–2021. CIR, crude incidence rate; CMR, crude mortality rate; CDR, crude disability-adjusted life years rate; ASIR, age standard incidence rate; ASMR, age standard mortality rate; ASDR, age standard disability-adjusted life years rate; YLLS, years of life lost; YLDs, years lived with disability; DALYs, disability-adjusted life years. **(A)** Incidence; **(B)** mortality; **(C)** YLLs; **(D)** YLDs; **(E)** DALYs **(J)** 0–4 years YLDs; **(K)** 5–9 years YLDs.

**Figure 2:**
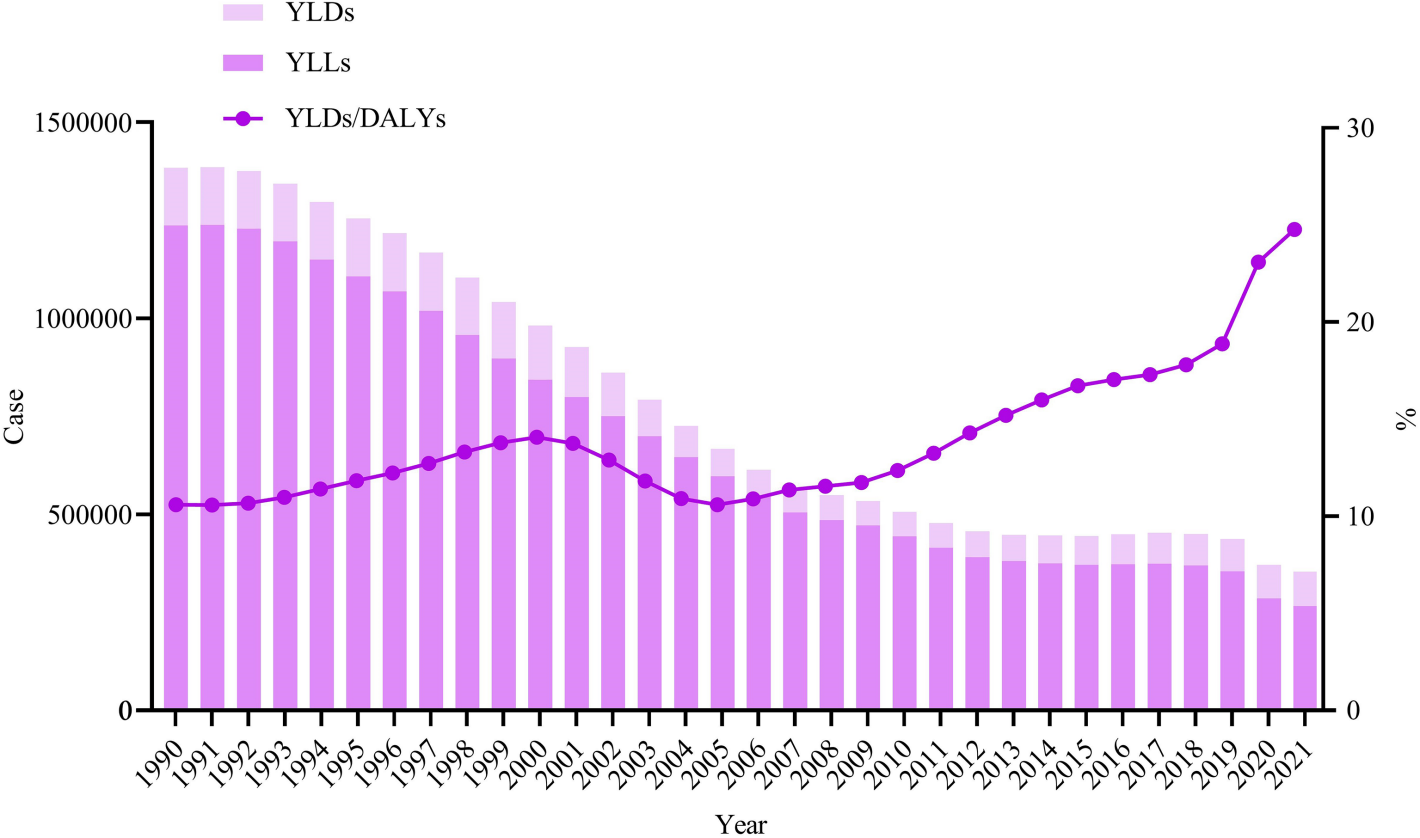
Changes in YLLs and YLDs among children aged 0–14 years in China, 1990–2021. YLLs, years of life lost; YLDs, years lived with disability; DALYs, disability-adjusted life years.

Between 1990 and 2021, children aged 0–14 years caused 163,769,426 incident cases and 255,840 deaths, resulting in a total of 25,085,796 DALYs. The ASIR declined from 2196.16/100,000 in 1990 to 1985.72/100,000 in 2021, representing a decline of 9.58%. Similarly, the ASMR, age-standardized YLLs rate, age-standardized YLDs rate, and ASDR all exhibited a declining trend, with declines of 72.03, 72.48, 29.68, and 67.95%, respectively (Supplementary Table 1; Figure 1). Regarding gender differences, both males and females experienced a similar declining trend (Figure 1). Additionally, although the absolute numbers and rates of YLLs and YLDs declined over time, the proportion of YLDs relative to DALYs has been consistently increased from 1990 to 2021 (*χ*_trend_^2^ = 99,357.261, *p* < 0.001; Figure 2).

### Temporal trends in disease burden of falls among children aged 0–14 years in China, 1990–2021

**Figure 3:**
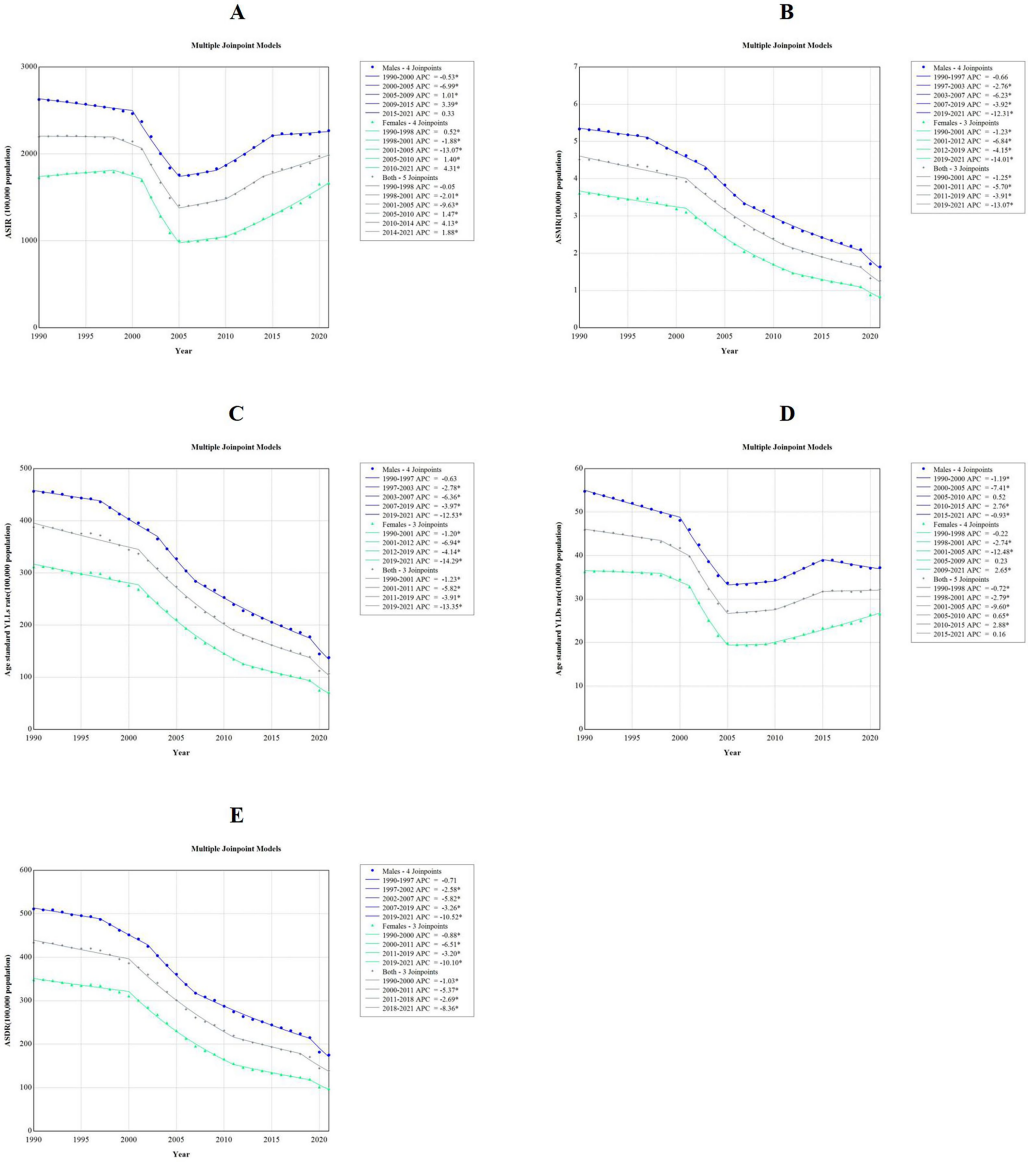
Trends in disease burden of falls among children aged 0–14 years by sex: a joinpoint regression model from 1990 to 2021. APC, annual percentage change; YLLs, years of life lost; YLDs, years lived with disability; DALYs, disability-adjusted life years; ASIR, age standard incidence rate; ASMR, age standard mortality rate; ASDR, age standard disability-adjusted life years rate. **(A)** ASIR; **(B)** ASMR; **(C)** age standard YLLS rate; **(D)** age standard YLDs rate; **(E)** ASDR.

Between 1990 and 2021, the ASIR exhibited a downward trend, with an AAPC of −0.34% (95%*CI*: −0.39% to −0.28%, *p* < 0.001). Similarly, the ASMR, age-standardized YLLs rate, age-standardized YLDs rate, and ASDR all demonstrated a downward trend, with AAPCs of −4.18% (95%*CI*: −4.37% to −4.06%, *p* < 0.001), −4.24% (95%*CI*: −4.43% to −4.11%, *p* < 0.001), −1.16% (95%*CI*: −1.22% to −1.11%, *p* < 0.001), −3.68% (95%*CI*: −3.80% to −3.57%, *p* < 0.001), respectively. Regarding gender differences, both males and females experienced similar downward trends in the ASIR, ASMR, age-standardized YLLs rate, age-standardized YLDs rate, and ASDR. Notably, upward trends were observed in the ASIR during two distinct periods: from 2010 to 2014, with an APC of 4.13% (95%*CI*: 3.38 to 5.07%, *p* < 0.001) and from 2014 to 2021, with an APC of 1.88% (95%*CI*: 1.44 to 2.18%, *p* < 0.001). Additionally, the age-standardized YLDs rate also showed upward trends during the periods from 2010 to 2015, with an APC of 2.88% (95%*C*I: 2.36 to 3.89%, *p* < 0.001) and from 2015 to 2021, with an APC of 0.16% (95%*CI*: −0.28 to 0.52%, *p* = 0.358). However, among males, a downward trend was observed from 2015 to 2021, with an AAPC of −0.93% (95%*CI*: −1.29% to −0.59%, *p* < 0.001; Figure 3, Supplementary Tables 2–6).

**Figure 4:**
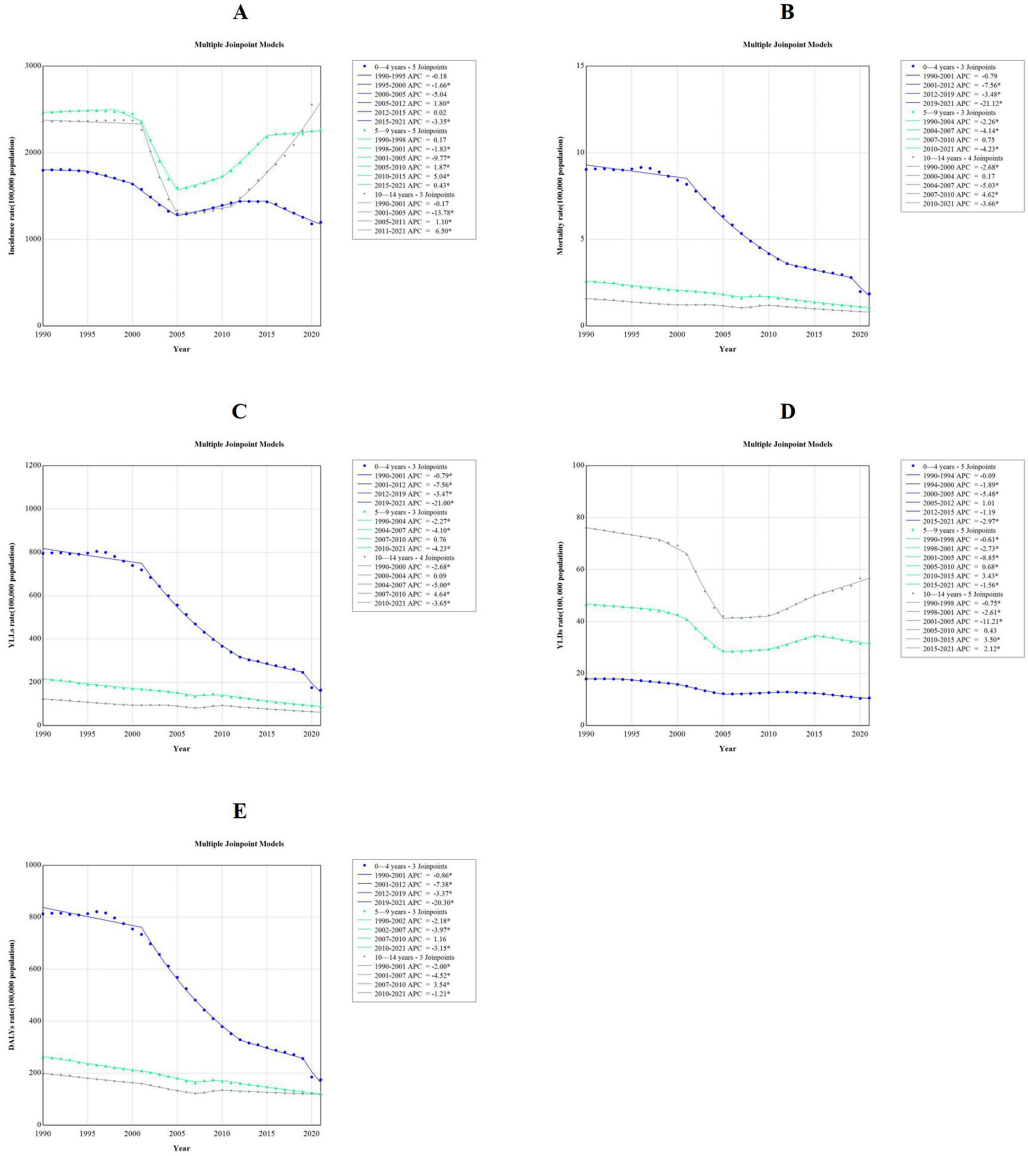
Trends in disease burden of falls among children aged 0–14 years by agegroup: a joinpoint regression model from 1990 to 2021. AРС, annual percentage change; YLLS, years of life lost; YLDs, years lived with disability; DALYs. disability-adjusted life years. **(A)** Incidence rate; **(B)** mortality rate; **(C)** YLLs rate; **(D)** YLDs rate; **(E)** DALYs rate.

With respect to age-specific trends, children aged 0 to 4 years exhibited a downward trend in the ASIR, ASMR, age-standardized YLLs rate, age-standardized YLDs rate, and ASDR, with AAPCS of −1.38% (95%*CI*: −1.46 to −1.29, *p* < 0.001), −5.26% (95%*CI*: −5.60 to −5.00, *p* < 0.001), −5.25% (95%*CI*: −5.58 to −4.99, *p* < 0.001), −1.75% (95%*CI*: −1.83 to −1.67, *p* < 0.001), −5.13% (95%*CI*: −5.46 to −4.88, *p* < 0.001), respectively. Similarly, children aged 5 to 9 years and 10 to 14 years also experienced downward trends in these metrics from 1990 to 2021. However, children aged 10 to 14 years exhibited an upward trend in incidence rate from 1990 to 2021, with an AAPC of 0.27% (95%*CI*: 0.16 to 0.38%, *p* < 0.001; Figure 4, Supplementary Tables 7–11).

### Projection of the disease burden of falls among children aged 0–14 years

**Figure 5:**
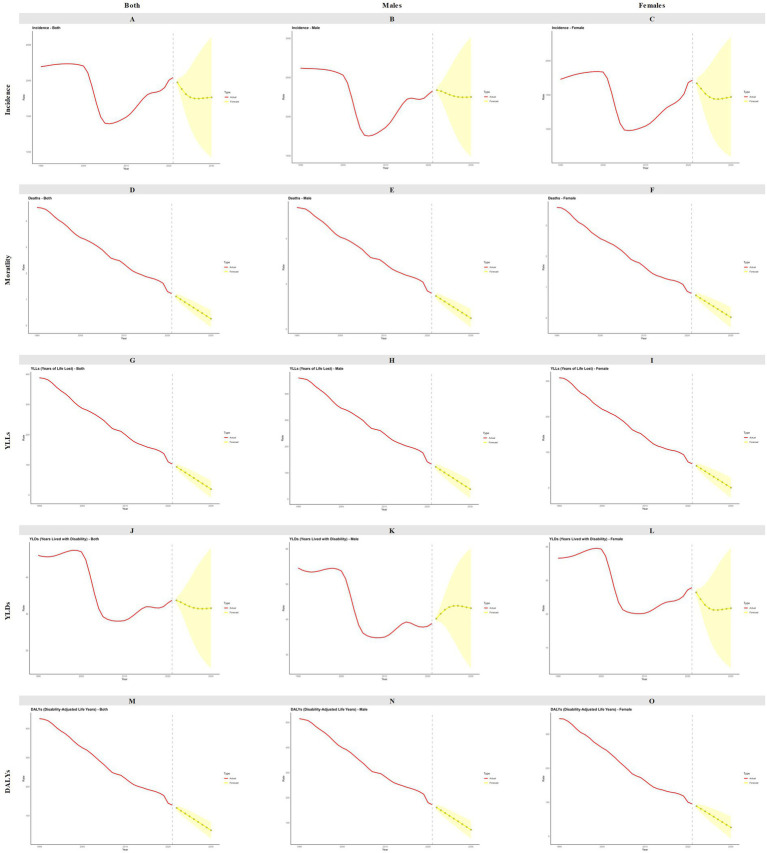
Forecast for incidence, mortality, YLLs, YLDs, DALYs to 2030 among children aged 0 to 14 years in China by sex. YLLs, years of life lost; YLDs, years lived with disability; DALYs, disability-adjusted life years. **(A)** Both incidence; **(B)** males incidence; **(C)** females incidence; **(D)** both mortality: **(E)** males mortality; **(F)** females mortality; **(G)** both YLLs; **(H)** males YLLs; **(I)** females YLLs; **(J)** both YLDs; **(K)** males YLDs: **(L)** females YLDs; **(M)** both DALYs; **(N)** males DALYs; **(O)** females DALYs.

**Figure 6:**
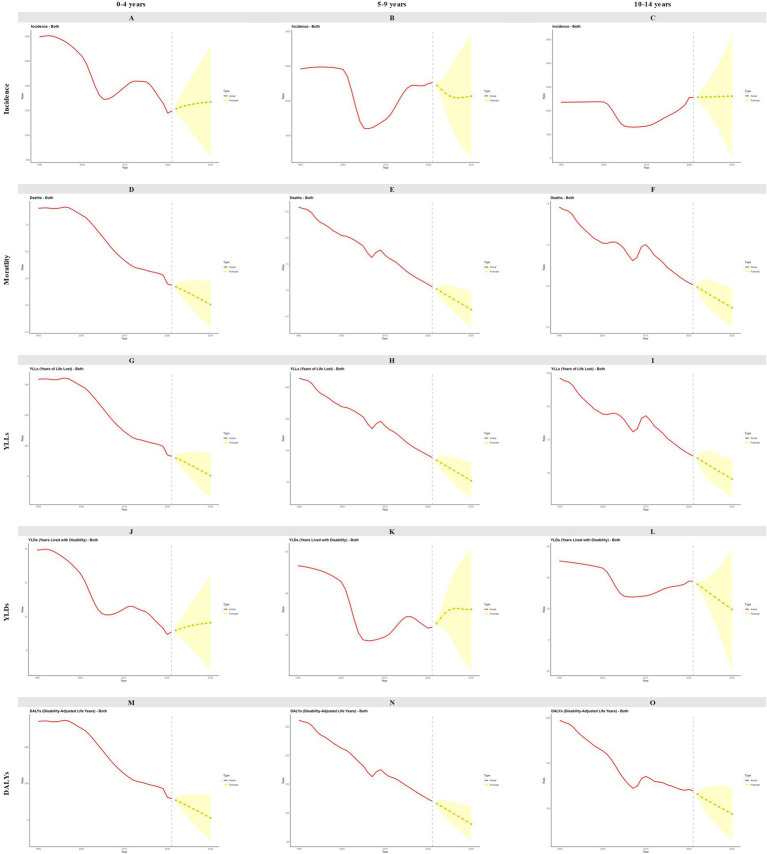
Forecast for incidence, mortality, YLLs, YLDs, DALYs to 2030 among children aged 0 to 14 years by age in China. YLLs, years of life lost; YLDs, years lived with disability; DALYs, disability-adjusted life years. **(A)** 0–4 years incidence; **(B)** 5–9 years incidence; **(C)**10–14 years incidence; **(D)** 0–4 years mortality; **(E)** 5–9 years mortality, **(F)** 10–14 years mortality, **(G)** 0–4 years YLLs; **(H)** 5–9 years YLLs; (1)10–14 years YLLs; **(J)** 0–4 years YLDs; **(K)** 5–9 years YLDs; **(L)** 10–14 years YLDs; **(M)** 0–4 years DALYs; **(N)** 5–9 years DALYs; **(O)** 10–14 years DALYs.

By 2030, the ASIR, ASMR, age-standardized YLLs rate, age-standardized YLDs rate, and ASDR are projected to continue declining. Similar trends are anticipated in both males and females. However, a notable exception is observed in the age-standardized YLDs rate among males, which is projected to increase by 2030 (Figure 5). In terms of age-specific disease burden, the mortality rate, YLLs, and DALYs are expected to continue their downward trend across different age groups. Conversely, the incidence among children aged 0–4 years is projected to rise, and an increase in YLDs is anticipated among children in both the 0–4 years and 5–9 years age groups (Figure 6). The predictive performance of ARIMA model was shown in (Supplementary Tables 12, 13).

## Discussion

To our knowledge, this is the first comprehensive evaluation of the disease burden associated with falls among children aged 0–14 years in China over the period 1990–2021. A longitudinal analysis of GBD data over the past 32 years revealed key trends in the burden of falls among this population. Although the ASIR, ASMR, age-standardized YLLs, age-standardized YLDs, and ASDR all demonstrated declining trends, stratified analysis revealed that male children in China consistently exhibited higher ASIR, ASMR, age-standardized YLLs, age-standardized YLDs, and ASDR from falls compared with females. Mortality peaked among children aged 0–4 years, whereas falls incidence remained relatively high among children aged 5–9 years. Moreover, falls incidence and YLDs rates showed an upward trend among children aged 10–14 years. The current study aims to contextualize these results, evaluate implications for policy and practice, suggest directions for future research.

From 1990 to 2021, the burden of falls among children aged 0 to 14 years in China exhibited a fluctuating decline and five significant segments were identified between 1990 and 2021. Joinpoint regression model revealed a sustained decline in the ASIR of falls among Chinese children aged 0–14 years across three successive segments: 1990–1998, 1998–2001 and 2001–2005, with the steepest reduction occurring after 2001. This downward trend coincided with progressive legislative and policy reforms (9), most notably the Law on the Protection of Minors (1991), which codified the prevention of unintentional childhood injuries, and the National Program for Child Development in China (2001–2010), which established a mandatory 30% reduction target for child-injury mortality and catalysed nationwide, multi-sectoral interventions targeting building safety, toy standards and recreational-facility management. On the other hand, the observed trend was also attributable to improvements in medical standards, expanded coverage of basic public health services, and heightened public awareness of injury prevention (20). Furthermore, ongoing improvements in infrastructure, such as smoother roads and the installation of guardrails in public spaces, had created safer environments for children to play, thereby reducing the incidence of falls (1).

However, Joinpoint regression model revealed that the ASIR of falls among children aged 0–14 years in China showed an upward trend from 2005 to 2021, the ASIR reversed and rose sharply. This inflection was attributable, at least in part, to enhanced ascertainment following the expansion of China’s national injury-surveillance system to the 0–14 age group, which improved case capture. Furthermore, children’s activity spaces and environments had become increasingly complex with urbanization accelerating, potentially leading to heightened risks of falls, particularly falls from height. Higher levels of screen time were associated with an increased risk of falls, potentially mediated by insufficient sleep and diminished attention (21). Insufficient physical activity was associated with delayed development of balance and muscle strength, thereby increasing falls risk (22). Engaging in moderate physical activity improved bone mineral density, muscular performance, and sensorimotor control, thereby significantly reducing falls incidence (23). However, high-intensity physical activity could induce neuromuscular fatigue and extend environmental exposure, thereby paradoxically elevating falls risk (24). Therefore, maintaining an optimal volume of physical activity reduces falls incidence by enhancing neuromuscular control, bone strength, and protective fall-response skills. The impact of school-based physical-activity policy changes on childhood falls is complex. A study from Beijing in 2022 revealed that daily moderate-to-vigorous physical activity among primary-school pupils increased by 12 min, concomitant with a 1.3‰ reduction in clinic visits for fall-related fractures (25). However, 40.4% of falls among children aged 6–17 years in China occur on school premises (12). Consequently, school-based fall-risk reduction is maximized when physical-activity policies simultaneously incorporate (1) increased activity time, (2) teacher–student fall-prevention education, and (3) environmental safety modifications.

Since 2014, the upward momentum had attenuated, the slope flattened significantly. This deceleration aligned temporally with the rollout of the Healthy China 2030 campaign, which prioritised evidence-based, region-specific fall-prevention strategies for children and thereby mitigated previously escalating risks. The incidence of falls had been on the rise in recent years, indicating gaps in children’s safety education that leave them inadequately aware of falls risks. Concurrently, new safety hazards may emerge in home and community environments, such as the proliferation of high-rise buildings and inconsistent safety standards for playground equipment, all of which heightened the risk of childhood falls.

Given the multifactorial etiology of childhood falls, enhanced surveillance and in-depth analysis are imperative to identify the specific factors contributing to the observed increasing trend. It is essential to develop targeted interventions, which may encompass improving urban planning to create safer environments, strengthening safety education to raise awareness of fall risks among children and caregivers, and enhancing safety standards in homes and communities, particularly with regard to the design and maintenance of playground equipment and the implementation of fall-prevention measures in high-rise buildings. For example, when developing intervention measures for child falls in playgrounds, it is essential to consider factors such as the type of facility, potential injury severity, cost-effectiveness, inspection frequency, and responsible entities. Additionally, practical implementation in both urban and rural areas of China must be thoroughly evaluated to propose tailored interventions (Supplementary Table 14).

The effectiveness of these interventions must be continuously evaluated through rigorous monitoring and feedback mechanisms, with adjustments made based on actual outcomes to ensure effective prevention of childhood falls. Additionally, the YLDs among children aged 0–14 years increased rapidly from 2005 to 2015, this observed reversal trend may be attributed to progressive enhancements in the completeness, coverage and coding quality of China’s national Disease Surveillance Points (DPS) system over the study interval. From 2002 to 2012, The DSP system was included 161 surveillance points, distributed across all 31 provinces in China, which ensured that the monitored population exceeded 77 million individuals, accounting for approximately 6% of the national population. Since 2012, the Chinese government has integrated and expanded the original Ministry of Health’s cause-of-death statistical system, the National Disease Surveillance System, and other death reporting systems to establish a nationally representative surveillance framework. Following this integration, the number of monitoring points has been increased to 605, with a monitored population exceeding 300 million individuals. This expanded system now covers approximately 24% of the national population. Then, the trends of YLDs remained relatively stable from 2015 to 2021. This trend changes also suggests that, despite improvements in medical care, the accessibility and quality of rehabilitation services may remain inadequate. Uneven distribution of medical resources across regions and populations has resulted in some children failing to receive timely and effective rehabilitation treatment after falls, thereby increasing their risk of long-term disability.

Gender-stratified analysis indicated that over the past 32 years, male children in China consistently exhibited higher ASIR, ASMR, age-standardized YLLs, age-standardized YLDs and ASDR from falls compared to female children. This finding was consistent with a study from Iran (26). Several factors may explain these gender disparities in the burden of falls among children aged 0–14 years. First, behavioral differences between male and female children play a significant role. Male children typically exhibit a higher propensity to engage in risk-taking behaviors and physical activities that increase the likelihood of falls. Second, prevailing gender norms in play behavior may contribute to differential exposure to fall-related risks, with male children more frequently encouraged to engage in adventurous or physically challenging play compared to their female counterparts (27, 28). Third, male children may have greater exposure to environments with higher falls risks, such as construction sites, taller structures, and less supervised play areas. This increased exposure can lead to a higher incidence of falls (29). Fourth, differentiated approaches to guardianship may significantly influence the level of supervision and protection provided to male and female children. In many cultures, male children are often granted more freedom to explore their environment than female children. This cultural tendency can result in male children having greater autonomy and access to potentially hazardous areas, thereby increasing their risk of falls (12). Gender-specific interventions are essential and should be implemented in the following three levels: (1) family level: parents should enhance supervision of young male children, particularly during solitary activities. Simultaneously, improve the home environment by installing safety gates, using childproof locks, and implementing other protective measures; (2) school level: schools should implement safety education programs to increase the safety awareness of male students. Additionally, the school environment should be made safer by installing protective measures, such as handrails on staircases and guardrails in corridors; (3) community level: communities should organize safety awareness campaigns to raise safety consciousness among parents and residents. Concurrently, the community environment should be improved, for example by installing guardrails in public areas and placing safety warning signs.

Age-stratified analysis indicated that the incidence rates, mortality rates, YLLs, YLDs, and DALYs among children aged 0–14 years in China showed a declining trend for those aged 0–4 years, 5–9 years, and 10–14 years from 1990 to 2021. However, the incidence of falls among children aged 10–14 years exhibited an upward trend (AAPC = 0.27%), particularly with a rapid increase during the period from 2011 to 2021 (APC = 6.50%). Additionally, the YLDs for children aged 10–14 years also showed an upward trend from 2010 to 2021. Despite the progress made in the overall prevention and control of childhood falls, the 10–14 year faces new challenges and risk factors related to falls. These emerging issues necessitate focused attention and the development of targeted preventive measures. Falls among children aged 10 to 14 primarily occured at school (12). Schools should enhance safety awareness education for students by integrating fall prevention knowledge and skills into physical education classes, health education sessions, and themed class meetings. This integration will help to raise students’ self-protection awareness and reduce the incidence of falls. Additionally, the widespread use of electronic devices may distract children during use, thereby increasing the likelihood of falls (30). Children aged 10–14 years exhibit greater mobility and independence, often engaging more in outdoor activities and sports, which increases their risk of falls.

Research conducted in multiple provinces and cities across China had indicated that falls were a leading cause of death among children (12, 31). These findings highlighted the significant impact of falls on children mortality and underscored the need for targeted prevention measure. The highest mortality rate from falls occurred among children aged 0 to 4 years, resulting in the highest YLLs for this age group. Children aged 0–4 years were in the infant and toddler stage, characterized by a lack of awareness of dangers and limited ability to protect themselves. Falls in this age group were more likely to result in head injuries. Infants and toddlers aged 0–4 years typically had relatively large head-to-body ratios, which made them more susceptible to head injuries during falls, as their larger heads can disproportionately affect their balance and increase the likelihood of head-first impacts. However, head injuries in children were often more easily detected and treated promptly due to their visible nature. Caregivers and healthcare providers are more likely to recognize and address head injuries quickly, resulting in higher reporting rates and more comprehensive treatment. Prompt detection and treatment can also contribute to the YLLs associated with this age group, as head injuries can be particularly severe and may have long-term consequences if not managed appropriately. Overall, the incidence rate of falls among children aged 5–9 years was relatively high. Falls in this age group primarily occurred in schools, kindergartens, or childcare facilities (12). Children aged 5–9 years begun to engage in outdoor activities independently, often without adult supervision and generally lack experience in distinguishing safety from hazardous situations. They are prone to encountering risks without recognizing them, and their limited self-control abilities make them susceptible to slips and falls during play. Simultaneously, parents may have insufficient awareness or fail to provide adequate supervision. Facilities within homes, schools, and communities may fail to keep pace with evolving activity needs of children. Therefore, comprehensive preventive measures must be implemented across four levels, family, school, community, and society to further reduce the injury and disease burden caused by falls among children aged 5–9 years.

Predictive analysis indicates that the incidence of falls among children aged 0–4 years is expected to increase, while YLDs for those aged 0–9 years are also projected to rise. This marked growth underscores the imperative for comprehensive and tailored prevention and management strategies. Studies had indicated that falls among children aged 0–4 years primarily occur within the home, suggesting that falls prevention efforts for this age group should focus on enhancing caregivers’ awareness of protective measures and fostering their ability to implement correct behavioral skills. Creating a safe home environment and strengthening effective supervision of children are essential components of a comprehensive prevention strategy (32). Encouraging behaviors, such as never leaving children unattended at heights and minimizing the use of baby walkers are also important steps in preventing falls (1). For children aged 5 to 9, falls prevention and control efforts should be implemented across family, school, community, and society.

The rising trend of years lived with disability (YLDs) in children aged 0–9 years underscores the urgent need to mitigate the burden of early-life impairment. First, expand infrastructure. The governments should prioritize the construction of county-level pediatric rehabilitation centres and dedicated departments within general hospitals, especially in underserved regions, to guarantee geographically equitable access to evidence-based rehabilitation for disabled children. Second, legislate mandatory safety audits. Education authorities, in conjunction with market-supervision, health, and emergency-management agencies, should embed compulsory annual safety assessments of kindergarten play facilities into the implementing rules of either the Preschool Education Law or the Work-Safety Law. Third, strengthen workforce capacity. Establish a certified cadre of rehabilitation instructors who deliver structured, home-based training to families, and develop accredited training packages on “Early Identification and Intervention of Childhood Disability” for parents, childcare staff, and preschool teachers. Fourth, improve financing and service integration. Include pediatric rehabilitation in the national health-insurance benefit package and create inter-sectoral mechanisms that seamlessly link medical rehabilitation with special-education services, thereby providing continuous, comprehensive support for children with disabilities.

To our knowledge, this study constitutes the first comprehensive trend analysis of disease burden among Chinese children aged 0–14 years, utilizing GBD data, providing both projections of future trends and novel epidemiological insights into the burden of falls among Chinese children. However, several limitations should be acknowledged. First, although the GBD database has enabled our analysis of trends in the burden of falls among children aged 0 to 14 years in China, a critical limitation that must be acknowledged is the absence of formal validation of the GBD estimates against China’s national injury-surveillance data. Subsequent studies should therefore prioritise triangulation of GBD outputs with high-resolution, multi-source repositories, most notably the China National Injury Surveillance System (CNISS) and standardised regional hospital records. Cross-validating these disparate data streams is indispensable for refining model parameterisation and ensuring that derived estimates faithfully capture the true epidemiological landscape of childhood falls in China and to enhance their utility for informing local public health policies. Second, GBD-China inputs are overwhelmingly derived from decennial censuses, the Disease Surveillance Points (DSP) system and the CDC’s Cause-of-Death registry, all of which are facility-based and omit the large reservoir of mild-to-moderate falls that never reach a clinic or hospital (33). Additionally, primary healthcare institutions hampered by delayed notification and systematic under-recording of non-fatal events, especially when external-cause coding is incomplete (34). Rural and remote DSP sites remain sparse and the paucity of numerator cases forces DisMod-MR to borrow strength across regions and impute values that can be markedly lower than the true population rates. The resulting shortfall in incident cases propagates directly into the YLD calculations, yielding a systematic downward bias in the national disability estimate. To establish an epidemiologically credible baseline for precision prevention, China urgently needs a multi-source enrichment strategy that couples hospital electronic medical-record text-mining with active, community-based follow-up and systematic linkage to school, ambulance and insurance databases. Third, beyond gender and age, additional factors such as urban–rural disparities, location, and timing of falls occurrence warrant consideration. Our study relies heavily on modeling processes, and model selection and parameter settings may influence the results. Fourth, due to temporal lags in global GBD data, our analysis is restricted to the time spanning 1990–2021. At last, the current study is a secondary analysis of the GBD 2021 datasets and therefore inherits the intrinsic limitations of the GBD study. This includes potential biases and inaccuracies in the original data sources and modeling processes that may affect the precision of our estimates. However, the GBD 2021 study employs robust statistical methods to mitigate these issues and produce the best possible estimates (35). Future studies should mitigate these limitations through incorporating more granular data and refining modeling techniques to provide a more nuanced comprehension of the epidemiology of falls among children.

The findings of current study provide valuable evidence to inform policy-making for falls prevention among Chinese children. Given that the burden of falls varies by gender and age, comprehensive interventions should be implemented targeting different genders and age and characteristics of falls occurrence. Injury prevention for falls among children is a systemic effort that requires government leadership, coordinated collaboration among relevant departments, and participation from society, schools, and families. Multi-channel health education should be implemented to enable early intervention, enhance children’ self-protection awareness and effectively reduce injury risks while promoting healthy development.

## Conclusion

The burden of falls remains a major public challenge among children aged 0–14 years in China, both in the present and across projected future trends. Mitigating this burden requires critical investments in further studies, the formulation of effective falls prevention strategies, and enhanced access to healthcare. Comprehensive and targeted interventions are essential to mitigate the risks and reduce the incidence of falls. These efforts should be coordinated across multiple sectors, including government, healthcare, education, and community organizations, to ensure a holistic approach to child safety. By enhancing awareness, implementing evidence-based prevention strategies, and providing timely and effective medical care, the burden of falls can be significantly reduced, thereby promoting the healthy development of children and adolescents in China.

## Data Availability

The datasets presented in this study can be found in online repositories. The names of the repository/repositories and accession number(s) can be found at: https://vizhub.healthdata.org/gbd-results/.

## References

[1] Sui L Lv Y Feng KX Jing FJ. Burden of falls in China, 1992-2021 and projections to 2030: a systematic analysis for the global burden of disease study 2021. Front Public Health. (2025) 13:1538406. doi: 10.3389/fpubh.2025.1538406, 40190758 PMC11968356

[2] Jiang H Sun N Yang H Li Y Jiang L Zhao H . Global, regional, and national time trends in falls and their predictions: an age-period-cohort analysis of the global burden of disease study 2021. Front Public Health. (2025) 13:1598507. doi: 10.3389/fpubh.2025.1598507, 40823243 PMC12354658

[3] Li C Jiao J Hua G Yundendorj G Liu S Yu H . Global burden of all cause-specific injuries among children and adolescents from 1990 to 2019: a prospective cohort study. Int J Surg. (2024) 110:2092–103. doi: 10.1097/JS9.0000000000001131, 38348839 PMC11020088

[4] Park H Kang H. Incidence of falls and fall-related characteristics in hospitalized children in South Korea: a descriptive study. Child Health Nurs Res. (2024) 30:176–86. doi: 10.4094/chnr.2024.016, 39081183 PMC11294899

[5] GBD 2016 Traumatic Brain Injury and Spinal Cord Injury Collaborators. Global, regional, and national burden of traumatic brain injury and spinal cord injury, 1990-2016: a systematic analysis for the global burden of disease study 2016. Lancet Neurol. (2019) 18:56–87. doi: 10.1016/S1474-4422(18)30415-0, 30497965 PMC6291456

[6] Pomerantz WJ Gittelman MA Hornung R Husseinzadeh H. Falls in children birth to 5 years: different mechanisms lead to different injuries. J Trauma Acute Care Surg. (2012) 73:S254–7. doi: 10.1097/TA.0b013e31826b017c, 23026963

[7] Er Y Duan L Wang Y Ji C Gao X Deng X . Analysis on data from Chinese National Injury Surveillance System, 2008-2013 on the characteristics of falls. Zhonghua Liu Xing Bing Xue Za Zhi. (2015) 36:12–6. doi: 10.3760/cma.j.issn.0254-6450.2015.01.004 25876857

[8] National Center for Chronic and Non-communicable Diseases Prevention and Control, Chinese Center for Disease Control and Prevention, National Health Commission Statistics and Information Center. The Chinese cause of death surveillance dataset (edition 2018). Beijing: China Science and Technology Press (2019).

[9] Zhang MG Zhou YB Li CC Qu MB Meng JJ Cai Q . Levels and trends of significant injury-caused deaths in the Chinese population, 2010-2019. Zhonghua Liu Xing Bing Xue Za Zhi. (2022) 43:871–7. doi: 10.3760/cma.j.cn112338-20220108-00015, 35725344

[10] National Center for Chronic and Non-communicable Diseases Prevention and Control, Chinese Center for Disease Control and Prevention. Retrospective report on the epidemiological status of child injuries in China (2016–2018). Beijing: China Population Press (2021).

[11] Lin WQ Lin L Yuan LX Pan LL Huang TY Sun MY . Association between meteorological factors and elderly falls in injury surveillance from 2014 to 2018 in Guangzhou, China. Heliyon. (2022) 8:e10863. doi: 10.1016/j.heliyon.2022.e10863, 36254282 PMC9568828

[12] Lu Z Ye P Wang Y Duan L Er Y. Analysis of the characteristics of falls among Chinese primary and middle school students in 2018. Chin J Sch Health. (2021) 42:917–21. doi: 10.16835/j.cnki.1000-9817.2021.06.028

[13] Wu Y Su B Gao J Zhong P Zheng X. Trends of falls mortality among older adults in urban and rural China, 1987-2021. Inj Prev. (2024):ip-2023-045225. doi: 10.1136/ip-2023-045225, 39002974

[14] Zhang YK Wang JX Ge YZ Wang ZB Zhang ZG Zhang ZW . The global burden of vertebral fractures caused by falls among individuals aged 55 and older, 1990 to 2021. PLoS One. (2025) 20:e0318494. doi: 10.1371/journal.pone.0318494, 40198621 PMC11978109

[15] GBD 2021 Diseases and Injuries Collaborators. Global incidence, prevalence, years lived with disability (YLDs), disability-adjusted life-years (DALYs), and healthy life expectancy (HALE) for 371 diseases and injuries in 204 countries and territories and 811 subnational locations, 1990-2021: a systematic analysis for the global burden of disease study 2021. Lancet. (2024) 403:2133–61. doi: 10.1016/s0140-6736(24)00757-8 38642570 PMC11122111

[16] Tu X Zhang F Chen J Tang M. Down syndrome burden in China and globally: a comparative analysis of 1990-2021 trends and future projections based on GBD 2021 database. Front Public Health. (2025) 13:1632250. doi: 10.3389/fpubh.2025.1632250, 40860557 PMC12375605

[17] Chen S Wang X Zhao J Zhang Y Kan X. Application of the ARIMA model in forecasting the incidence of tuberculosis in Anhui during COVID-19 pandemic from 2021 to 2022. Infect Drug Resist. (2022) 15:3503–12. doi: 10.2147/idr.S367528, 35813085 PMC9268244

[18] Xu M Tang C Shen Y Zhang Y Bao L. Analysis of the burden of intracerebral hemorrhage in the Asian population aged 45 and older and ARIMA model prediction trends: a systematic study based on the GBD 2021. Front Neurol. (2025) 16:1526524. doi: 10.3389/fneur.2025.1526524, 40027166 PMC11869383

[19] Liu J Xie X Li Y Pu Y Li Y Yang L. Burden of autism spectrum disorder in Japan from 1992 to 2021 and its prediction until 2050: results from the GBD study. Front Psychol. (2025) 16:1619085. doi: 10.3389/fpsyt.2025.1619085, 40698055 PMC12279874

[20] Er YL Jin Y Ye PP Ji CR Wang Y Deng X . Disease burden on falls among 0-19 years old population in China, in 1990 and 2017. Zhonghua Liu Xing Bing Xue Za Zhi. (2019) 40:1363–8. doi: 10.3760/cma.j.issn.0254-6450.2019.11.005, 31838805

[21] Zhang R Zhu H Xiao Q Wu Q Jin Y Liu T . Association between excessive screen time and falls, with additional risk from insufficient sleep duration in children and adolescents, a large cross-sectional study in China. Front Public Health. (2024) 12:1452133. doi: 10.3389/fpubh.2024.1452133, 39712319 PMC11659216

[22] Willemse L Wouters EJM Bronts HM Pisters MF Vanwanseele B. The effect of interventions anticipated to improve plantar intrinsic foot muscle strength on fall-related dynamic function in adults: a systematic review. J Foot Ankle Res. (2022) 15:3. doi: 10.1186/s13047-021-00509-0, 35057831 PMC8772142

[23] Kwok WS Dolja-Gore X Khalatbari-Soltani S Byles J Oliveira JS Pinheiro MB . Physical activity and injurious falls in older Australian women: adjusted associations and modification by physical function limitation and frailty in the Australian longitudinal study on women's health. Age Ageing. (2023) 52:52. doi: 10.1093/ageing/afad108, 37389559 PMC10312133

[24] Mohd Safee MK Abu Osman NA. Correlation between postural stability and fall risk in trans-femoral amputees due to muscle fatigue. J Phys Ther Sci. (2024) 36:592–7. doi: 10.1589/jpts.36.592, 39354923 PMC11441891

[25] Chen L. (2022). “Research Progress of school sports policy in China.” in International Conference on Humanities and Education.

[26] Azarbakhsh H Jafari F Dehghani SS Hassanzadeh J Janfada M Mirahmadizadeh A. Trend analysis of unintentional fall mortality and years of life lost in the Fars Province of Iran, 2004-2019. Iran J Public Health. (2024) 53:1427–36. doi: 10.18502/ijph.v53i6.15916, 39430156 PMC11488564

[27] Peden M Ameratunga S Mytton J Vincenten J Wainiqolo I Puvanachandra P . The step safely guidelines: a catalyst to address the burden of falls in children and adolescents. Lancet Child Adolesc Health. (2022) 6:673–4. doi: 10.1016/S2352-4642(22)00194-8, 35810747

[28] Huebner CE Milgrom P. Evaluation of a parent-designed programme to support tooth brushing of infants and young children. Int J Dent Hyg. (2015) 13:65–73. doi: 10.1111/idh.12100, 25070036 PMC4486350

[29] Zhang K Qi J Zuo P Yin P Liu Y Liu J . The mortality trends of falls among the elderly adults in the mainland of China, 2013-2020: a population-based study through the National Disease Surveillance Points system. Lancet Reg Health West Pac. (2022) 19:100336. doi: 10.1016/j.lanwpc.2021.100336, 35257118 PMC8897056

[30] Pelicioni PHS Chan LLY Shi S Wong K Kark L Okubo Y . Impact of mobile phone use on accidental falls risk in young adult pedestrians. Heliyon. (2023) 9:e18366. doi: 10.1016/j.heliyon.2023.e18366, 37701410 PMC10493431

[31] Wei XL Du WC Wang R Zhou JY Yu H Lu Y . Epidemic characteristics and trend analysis of major injuries deaths among children and adolescents in Jiangsu Province from 2012 to 2021. Zhonghua Liu Xing Bing Xue Za Zhi. (2024) 45:536–41. doi: 10.3760/cma.j.cn112338-20230912-00150, 38678349

[32] Molocznik A Omaki E Wagner K Shields WC McDonald EM Solomon BS . “Before I could get him, he fell”: experiences, concerns, and fall prevention strategies of parents with young children. Clin Pediatr (Phila). (2023) 62:1426–34. doi: 10.1177/00099228231161018, 36919814

[33] Du WX Yu H Zhou JY Wu M. Characteristics and disease burden of injury among residents in Jiangsu Province, 2017. Shanghai Prev Med. (2021) 9:813–7. doi: 10.19428/j.cnki.sjpm.2021.20489

[34] Xu YQ Zhou CL Sun QL Jin DH Hu JX He GH . Association between temperature and injury death and related excess death burden in Hunan Province. J Environ Occup Med. (2025) 5:528–35. doi: 10.11836/JEOM24511

[35] Xiao Y Chen TT Zhang Z Liu L Du N. Changes in the disease burden of depressive disorders among middle-aged and older adults (aged 45-89) in China over 30 years: insights from the global burden of disease study 2021. Int J Geriatr Psychiatry. (2025) 40:e70069. doi: 10.1002/gps.70069, 40090859

[36] Lan ZJ Liu CH Wang HJ Wang YW Kan SH Jiao YL . Temporal trends in the burden of vertebral fractures caused by falls in China and globally from 1990 to 2021: a systematic analysis of the global burden of disease study 2021. Arch Public Health. (2025) 83:42. doi: 10.1186/s13690-025-01500-y, 39962620 PMC11831765

